# Rescinding Community Mitigation Strategies in an Influenza Pandemic

**DOI:** 10.3201/eid1403.070673

**Published:** 2008-03

**Authors:** Victoria J. Davey, Robert J. Glass

**Affiliations:** * Veterans Health Administration, Washington, DC, USA; † Uniformed Services University of the Health Sciences, Bethesda, Maryland, USA; ‡ Sandia National Laboratories, Albuquerque, New Mexico, USA

**Keywords:** Pandemic influenza, community mitigation, school closing, nonpharmaceutical interventions, social distancing, sequestering, quarantine, epidemic modeling, agent-based model, research

## Abstract

Using a networked, agent-based computational model of a stylized community, we evaluated thresholds for rescinding 2 community mitigation strategies after an influenza pandemic. We ended child sequestering or all-community sequestering when illness incidence waned to thresholds of 0, 1, 2, or 3 cases in 7 days in 2 levels of pandemic severity. An unmitigated epidemic or strategy continuation for the epidemic duration served as control scenarios. The 0-case per 7-day rescinding threshold was comparable to the continuation strategy on infection and illness rates but reduced the number of days strategies would be needed by 6% to 32% in mild or severe pandemics. If cases recurred, strategies were resumed at a predefined 10-case trigger, and epidemic recurrence was thwarted. Strategies were most effective when used with high compliance and when combined with stringent rescinding thresholds. The need for strategies implemented for control of an influenza pandemic was reduced, without increasing illness rates.

## Abstract 2

Thresholds for these strategies reduced the number of days strategies were needed without increasing illness rates.

Community goals during an influenza pandemic include protecting people from illness and maintaining critical societal functions by limiting time away from usual occupations. Vaccine and antiviral medications are standards of influenza prevention, postexposure prophylaxis, and treatment (*1*). However, vaccine for a new influenza subtype may not begin to be available for at least 20 weeks after the onset of a pandemic and would be supplied over many months. Antiviral drugs may be in greater supply, but their effectiveness and rapid availability are uncertain (*2*,*3*). The US government has proposed community mitigation strategies for limiting the harm or managing the pace of an influenza pandemic until vaccine becomes available. These behavioral- and pharmaceutical-based strategies rely on reducing viral transmission and include dismissing schools and public gatherings, voluntary sequestering in the home, staggering work shifts, keeping symptomatic persons isolated, and treating ill persons rapidly with antiviral drugs and providing antiviral prophylaxis for their household contacts. These community mitigation strategies would be applied according to a pandemic severity index (PSI) scaled as categories 1–5. Category 5 would be a 1918-like event (case-fatality rate >2.0%) and category 1 (case-fatality rate <0.1%) would be akin to a bad seasonal influenza year (*3*). Modeling studies have estimated the effectiveness of mitigation strategies with and without vaccine and antiviral drugs (*4*–*8*). However, an independent review of pandemic influenza modeling studies raised the question of whether and when community containment strategies might safely be rescinded without reinitiating an epidemic (*9*).

An earlier study of this computational model demonstrated that closing schools and curtailing contacts of children and teenagers for the duration of a mild 1957-like epidemic in a stylized community reduced the number of infected persons by >90% (*10*). The model was constructed with assumptions that children and teenagers are responsible for influenza transmission in a community because of the frequency and nature of their person-to-person contacts (*11*). However, sensitivity analyses showed that permutations of mitigation strategies that included adults were effective at reducing infections in the model population, even for more highly infective 1918-like viral strains or with removal of enhanced children/teenagers’ role in transmission (*10*). Several studies have shown that combining strategies such as social distancing of adult groups in and outside the workplace and removing symptomatic persons from community contact substantially reduced infections except in epidemics caused by the most infectious viral strains (*4*–*10*).

The US government’s community mitigation guidance recommends rapid initiation of strategies, then up to 4 weeks of school closure for a PSI 2–3 pandemic and 12 weeks for a PSI 4–5 pandemic. However, this guidance fails to address the gap between 12 weeks of mitigation strategies and estimated vaccine availability beginning at 20 weeks, especially if antiviral drugs were of limited effectiveness or availability (*3*). A pandemic could recur in the intervening period, and nonpharmaceutical community mitigation strategies with rules for their use would be valuable tools. Although nonpharmaceutical community mitigation measures have been used with apparent success in past pandemics (*12*,*13*), there are concerns about unintended consequences such as economic losses, interruption of education, and restrictions of personal freedom (*9*). The potential impact of community mitigation strategies warrants further study and consideration.

We evaluated effects of rescinding 2 community mitigation strategies for influenza pandemics, seeking a balance of the effect of illness, risk for epidemic recurrence, and minimization of the duration of mitigation strategies. The 2 strategies bracket mitigation measures that might be logically used in a situation in which effective vaccine and antiviral drugs are not available. The strategies are child sequestering, which is included in the US community mitigation guidance of February 2007, and a most conservative measure of all-community sequestering. We instituted strategies early, after 10 cases of mild (1957-like, PSI 1–2) or severe (1918-like, PSI 4–5) pandemic influenza occurred in a stylized community; these strategies were rescinded according to incident cases within a specific period.

## Methods

The model used in this study has been described (*10*). Briefly, it is a networked, agent-based computational model, Loki-Infect, developed at the National Infrastructure Simulation and Analysis Center, a collaborative center of Sandia National Laboratories and Los Alamos National Laboratories. Our model application simulates an influenza epidemic in a community of 10,000 populated according to the age breakdown of the 2000 US Census for a small US community (*14*). The community consists of 17.7% children 0–11 years of age, 11.3% teenagers 12–18 years of age, 58.5% adults 19–64 years of age, and 12.5% older adults >65 years of age.

The social contact network within the model determines how persons are linked so that transmission of influenza and its consequences may occur. Persons are placed in multiple groups that reflect their roles and functions within the larger social network. Groups include household settings (older adults, adults, teenagers, and children), school settings (teenagers, children), work settings (adults), and community settings (older adults, adults, teenagers, and children). Persons are further assigned to within-age group interactions to reflect routinely occurring social gatherings such as clubs and meetings. Within groups, persons are linked through an average number of person-to-person contact(s) per day on the basis of observations of behavior and activities of group members (*10*). Random links and contacts are built into the model to reflect unscheduled events such as chance face-to-face encounters.

Within the social contact network, each person in the community occupies 1 of 7 positions in the natural history of influenza (uninfected, latent infection, infectious presymptomatic, infectious symptomatic [20% circulating in the community, 80% diagnosed and staying home], infectious asymptomatic, immune, or dead). We also include an eighth position, a noninfectious recovery period (mean 7 days) for diagnosed persons to reflect expected illness caused by a pandemic strain. Opportunities for transmission within the network are selected stochastically and depend on multiple parameters, including infectivity of the virus, position of the person in the natural history of influenza, susceptibility of the person being infected, and infectiousness of the transmitting persons (*10*). Probabilities of progression through the natural history of influenza and susceptibilities follow current understanding of influenza infection, and reflect recent work of Ferguson et al. (*4*,*5*). When diagnosed and staying home, a person reduces contact frequency with all nonhousehold groups by a compliance level.

The 2 community mitigation strategies modeled were school closings with home sequestering of children and teenagers <18 years of age (hereafter called child sequestering) and home sequestering of all community members (hereafter called community sequestering). Child sequestering reduces contact frequencies for children and teenagers in school and all nonhousehold settings by a compliance level and doubles within-household contacts of children and teenagers. During child sequestering, 1 household adult stays home in households with children <11 years of age where they similarly adjust their group contact frequencies. Community sequestering reduces all nonhousehold contacts of all community members by a level of compliance and doubles within-household contacts. We considered compliance levels from 50% to 90% in increments of 10%. Adult days at home are a measure of the effect of the mitigation strategy and are counted as number of days adults are either sick at home, taking care of children, or sequestered themselves. Adult days at home in community sequestering are adjusted by compliance to reflect the percentage of time spent outside the home on a daily basis while the strategy is imposed.

Scenarios begin by infecting 10 adults chosen at random. Strategies are imposed after 10 persons within the community receive a diagnosis of influenza and end when the epidemic slows to the point that 0, 1, 2, or 3 newly diagnosed cases occur in 7 days (≈2 generation times of influenza [*5*]). Strategies are reimplemented if 10 new cases occur and are rescinded at the designated rescinding threshold. Control scenarios are an unmitigated base case (epidemics without mitigation strategies implemented) or continuation of strategies for the epidemic duration (child sequestering or community sequestering implemented at the 10–diagnosed case trigger and ended when the last incident case is recovered or dead).

We designed epidemic severity as a function of case-fatality rate and viral infectivity. We assumed a case-fatality rate of 2% of those with clinical illness. We applied 2 viral infectivities (I_D_) to yield 50% and 70% infection rates in the stylized community. Infection is defined as persons with viral infection with or without clinical illness. (Clinical illness rates are 50% of infection rates.) The 2 I_D_s result in reproduction numbers (R_0_, or the number of secondary cases produced by a source case in a susceptible population [*15*]) of ≈1.6 and 2.0, respectively. Thus, when the case-fatality rate is factored on 2 I_D_s, simulations yielded epidemics of 2 severities. The mild scenario resulted in a 1957-like epidemic classified by the US PSI as category 1–2. The severe scenario yielded a 1918-like epidemic of PSI 4–5 (*3*,*4*,*10*,*16*).

To capture the real-world heterogeneity of communities experiencing an epidemic, each scenario was simulated in 100 statistically identical networks of the community; each epidemic then propagated through the community stochastically. We include data only from simulations that resulted in epidemics (defined as >100 infected persons or 1% of the population). We report infection rates to emphasize that asymptomatic persons play a role in influenza transmission. We also report peak illness rates to quantify the effect of illness on the community. The Table lists study outcome measures and community epidemic management targets from the literature and our estimations of tolerable epidemic impact to the community (*17*).

**Table:** Epidemic management targets

Outcome	Target
No. epidemics*/1,070 simulations	Fewer than unmitigated base cases
Infection rate	<0.1 of population (n = 1,000)
Peak illness rate	<0.01 of population (n = 100)
Average no. cycles required	<2.0
Average days home per adult	Fewer than continuation strategies
Average no. days strategies imposed	Fewer than continuation strategies

*An epidemic is defined in the model as a simulation with a clinical illness rate >1% of the population.

## Results

**Figure 1:**
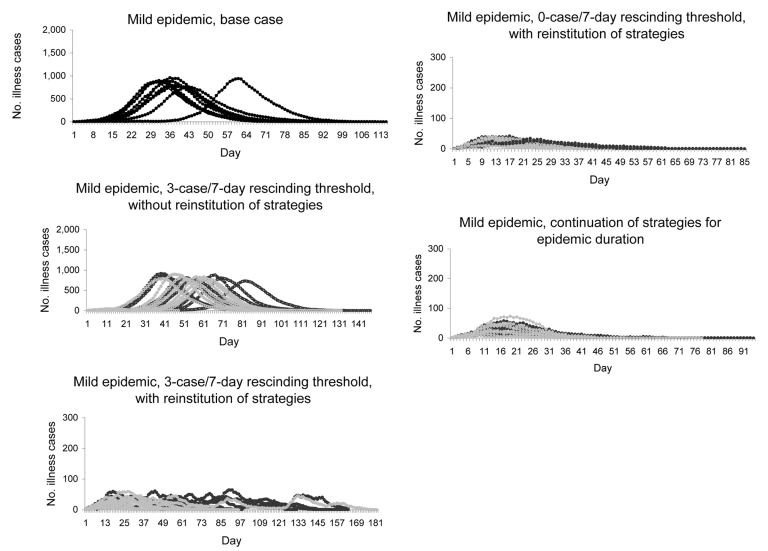
Mild epidemic (no. illness cases in a community of 10,000 by day) using 10 randomly selected simulations from 100 conducted for each scenario. Top panel shows unmitigated base case epidemic curves. Remaining panels show child sequestering strategy (dark lines) and community sequestering strategy (light lines). Each mitigation strategy is implemented at 90% compliance. (Note change in y-axis scale.)

**Figure 2:**
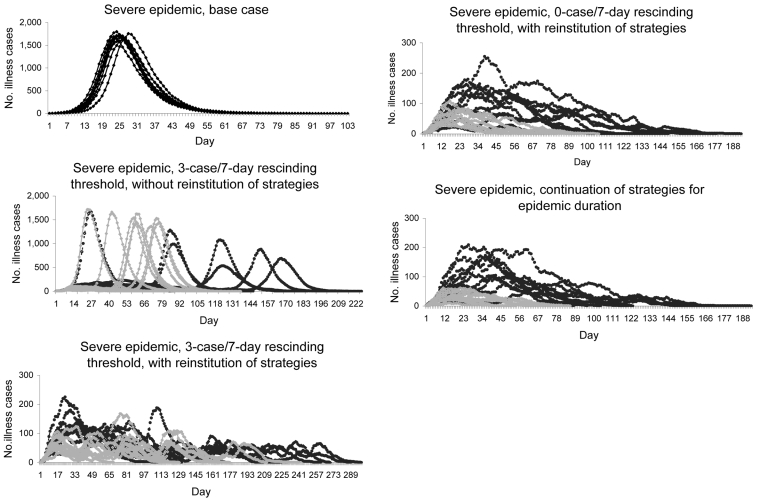
Severe epidemic (no. illness cases in a community of 10,000 by day) using 10 randomly selected simulations from 100 conducted for each scenario. Top panel shows unmitigated base case epidemic curves. Remaining panels show child sequestering strategy (dark lines) and community sequestering strategy (light lines). Each mitigation strategy is implemented at 90% compliance. (Note change in y-axis scale.)

We provide scenario outcomes for mild (Appendix Table 1) and severe (online Appendix Table, available from Appendix Table 2) epidemics that compare the unmitigated base case, continuation strategies, and rescinding thresholds of 0, 1, 2, or 3 cases in 7 days (hereafter rescinding thresholds assume cases/7 days). Results are displayed for 50%–90% compliance in 10% increments. We also provide epidemic curves for mild (Figure 1) and severe (Figure 2) epidemics beginning with unmitigated epidemics, then with rescinding thresholds under varying conditions, and ending with continuation strategy plots. Both mitigation strategies (child sequestering and community sequestering) are plotted on each graph.

### Unmitigated Base Case

In the mild, unmitigated base case epidemic, an average of 8.4% (840 cases) of the population was ill at peak. The combination of a mild epidemic and lack of strategy imposition meant that the average number of days adults spent at home either sick or tending sick children was 2 (range 0.4–2.4) over the course of the pandemic. In the severe epidemic, an average of 17.0% (1,700 cases) of the population was ill at peak, and adults spent an average of 3 (range 2.9–3.4) days at home (Appendix Table 2). Unmitigated base case epidemics showed a rapid onset, early peak (≈day 23 for mild or day 27 for severe) and resolution (most by day 100 for mild and day 80 for severe) (Figures 1, 2).

### Continuation of Strategies for the Epidemic Duration

Addition of mitigation strategies reduced peak illness rates (approximately an order of magnitude; Figures 1, 2). When child sequestering or community sequestering was continued until the last case was recovered or dead (continuation strategies), each equally controlled mild epidemics. For severe epidemics, infection and peak illness rates were higher for child sequestering than for community sequestering (peak illness rates were 1.4% [140 cases] for child sequestering and 0.7% [70 cases] for community sequestering at 90% compliance) (Appendix Tables 1, 2). Although each strategy reduced the peak illness rate by >90% from the unmitigated base cases, in severe epidemics, child sequestering did not meet epidemic control targets for infection rate and peak clinical illness attack rate; community sequestering was required to meet these targets (Table, Appendix Tables 1, 2). In contrast to the unmitigated base cases, adults were at home for longer periods with continuation strategies (12 days [mild] and 28 days [severe] for child sequestering; 43 days [mild] and 63 days [severe] for community sequestering; at 90% compliance) (Appendix Tables 1, 2).

### Rescinding Thresholds

Adding rescinding thresholds as a component of mitigation strategies resulted in several beneficial effects, influenced by whether a conservative or lax threshold was used and whether strategies were reinstituted if cases recurred. We assume 90% compliance with strategies; effects of lower compliance are considered in the next section.

Use of a 3-case threshold but not reinstituting strategies at the 10 new case trigger (Figures 1, 2) delayed the full epidemic by interjecting a preliminary period and peak where the epidemic is controlled by the mitigation strategy. After rescinding, a second 10-fold higher peak occurred (especially if child sequestering was used) and total illness attack rates approached those of the base case (Figures 1, 2). In comparison, by reinstituting cycles at the 10-case trigger even with the lax 3-case threshold, peak illness rates were dramatically flattened (to <100 cases for mild and generally <200 cases for severe epidemics), and the epidemic duration was lengthened because of the on-off cycling of mitigation strategies (Figures 1, 2). The 3-case threshold required an average of 3.2 (mild; community sequestering) to 5.8 cycles (mild; child sequestering) (Figures 1, 2, Appendix Tables 1, 2).

The 0-case threshold was comparable to continuation strategies in controlling infection and peak illness rates (Appendix Tables 1, 2, Figures 1, 2); <1.1 strategy cycles were needed to achieve control for both mild and severe epidemics. Infection and peak illness rates increased as the rescinding threshold was relaxed to 1, 2, or 3 cases (Appendix Tables 1, 2). The 0-case threshold decreased duration of epidemics when compared with the 3-case threshold. For example, in a severe epidemic, the maximum epidemic represented by these sample plots lasted 286 days with a 3-case threshold versus 169 days with a 0-case threshold (Figures 1, 2). Adult days at home in a mild epidemic were decreased from the continuation strategy by 25% (12 days to 9 days) for child sequestering and by 33% (43 days to 29 days) for community sequestering, both with the 0-case threshold. For a severe epidemic, adult days at home for child sequestering decreased less (28 to 27 days) with the continuation strategy than with community sequestering (63 to 49 days). Relaxing the rescinding threshold generally increased the number of adult days at home often above the continuation strategy (Appendix Tables 1, 2). Recurrence of epidemics was a function of rescinding threshold; an average of only 1/10 epidemics recurred for the 0-case threshold versus 10/10 for the 3-case threshold (for both mild and severe epidemics).

### Role of Compliance

**Figure 3:**
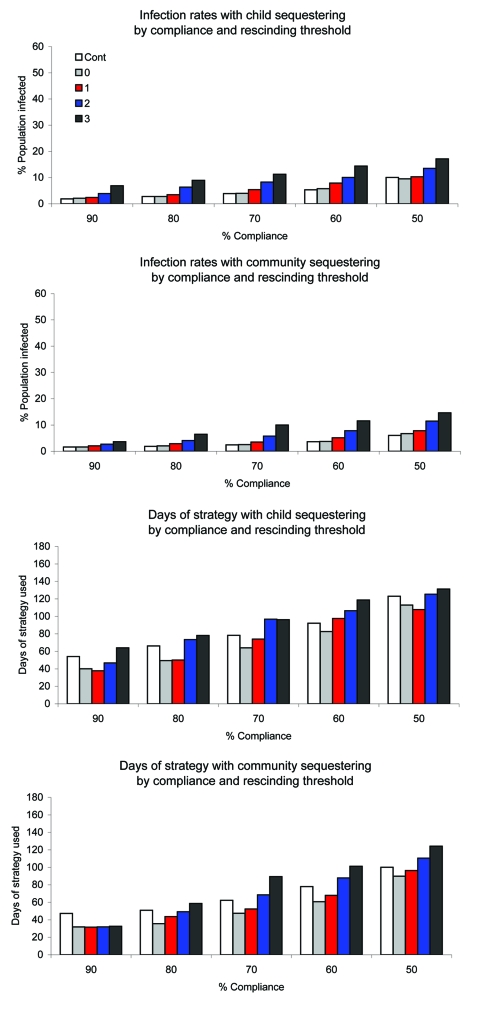
Infection rates and duration of strategy in mild epidemics by level of compliance for child sequestering and community sequestering, and rescinding threshold (Cont, 0, 1, 2, or 3). Cont, strategy continuation for the duration of the epidemic.

**Figure 4:**
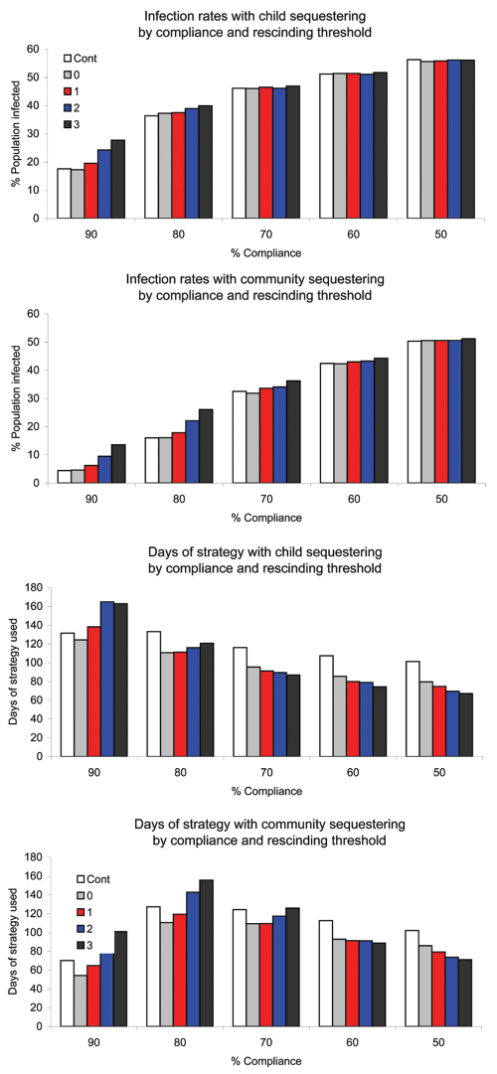
Infection rates and duration of strategy in severe epidemics by level of compliance for child sequestering and community sequestering, and rescinding threshold (Cont, 0, 1, 2, or 3). Cont, strategy continuation for the duration of the epidemic.

Compliance with imposed child sequestering or community sequestering could be <90% because of persons who provide essential community services, other needs for persons to circulate in the community, or fatigue with the strategy. Figures 3 and 4 compare infection rates and strategy time across the rescinding thresholds and levels of compliance.

For the control continuation strategies, reducing compliance with child sequestering or community sequestering eroded effectiveness in preventing epidemics and also affected infection and peak illness rates. For severe epidemics, high compliance was critical to epidemic control. When compliance decreased from 90% to 50%, infection rates tripled for child sequestering (from 17% to 56%) and increased 10-fold for community sequestering (5% to 50%) (Appendix Tables 1, 2). In contrast, because low compliance enabled the epidemic to pass through the community more quickly (infecting susceptible persons rapidly), adult days at home were fewer than at higher compliance (e.g., adult days at home in community sequestering were 51 days at 50% compliance and 63 days at 90% compliance (Appendix Tables 1, 2).

When rescinded at the 0-case threshold with 80%–90% strategy compliance, fewest days of strategies were needed (Appendix Tables 1, 2, Figures 3, 4). However, for severe epidemics, lower compliance (50%–70%) and the 0-case threshold used more strategy time than less restrictive ending case thresholds. Infection and peak illness rates showed little variation. In a severe epidemic with community sequestering implemented at 50% compliance, the infection rate was 50% with the 0-case threshold with 86 days of the strategy compared with an infection rate of 51% with the 3-case threshold with 71 days of strategy (Appendix Tables 1, 2). The model shows this result because once half the population is infected (and therefore immune), the epidemic R_0_ of 2.0 has effectively been halved. With fewer persons to infect at the end of the epidemic, use of the more conservative rescinding threshold has no effect on ultimate infection rates, requires additional days of strategy, and thus increases adult days at home.

## Discussion

We examined whether it was possible to safely rescind child sequestering and community sequestering in a waning mild (PSI 1–2) or severe (PSI 4–5) epidemic by using 0, 1, 2, or 3 new cases in 7 days as a threshold. Defined epidemic management targets reflected community goals aimed at 1) minimizing infection and peak illness rates (to reduce illness cases, transmission opportunities, and to limit healthcare surge); 2) minimizing days adults were kept from their usual occupations (to enable community functions to continue); and 3) minimizing local epidemic duration (to enable all community members to return to usual activities). Community mitigation strategies such as child sequestering and community sequestering may help achieve these goals. Ideally, strategies should be used for the minimum time necessary.

When modeled with the highest compliance and reinstitution of strategies in the event of epidemic recurrence, a rescinding threshold of 0 cases applied as a component of community sequestering contained PSI 1–2 or 4–5 epidemics with fewest days of mitigation strategy needed. Peak illness rates did not exceed 1% of the population and infection rates were <10%. The 0-case threshold applied as a component of child sequestering in severe epidemics did not control as well, but still substantially reduced infection and illness rates and shortened the number of days adults were required to be home and days strategies were used compared with continuing the strategies for the epidemic duration.

When less conservative rescinding thresholds of 1, 2, or 3 cases were used (strategies applied with high compliance), epidemic recurrences required multiple reinstitution of cycles, ultimately yielding marked increases in cases, number of adult days at home, and epidemic duration. Reducing compliance increased infection rates, peak illness rates, and adult days at home, and required duration of strategies. For severe epidemics and low compliance, epidemics were only marginally suppressed. Thus, relaxing ending thresholds to 3 cases did not add to the effect of illness and ultimately cost fewer days of strategies. These findings indicate that low compliance cannot be compensated for by a restrictive rescinding threshold. High compliance is the necessary enabler of successful mitigation strategies (not the rescinding threshold).

Reinstitution of strategies in the event of epidemic recurrence was necessary to prevent near base case levels of illness. Such reinstitution will be a critical action in an epidemic resurgence or in the event of a multiwave pandemic. The 1918 pandemic consisted of an apparent springtime herald wave followed by severe fall-winter waves, and a series of secondary waves lasting into 1920. Historical studies of the 1918 pandemic demonstrated that earlier initiation and longer duration of nonpharmaceutical measures were associated with lower peak and total death rates in US cities (*12*,*13*,*18*). However, pandemic-affected cities that instituted, then abandoned, mitigation strategies had more illness cases in subsequent pandemic waves (because more susceptible persons remained in the population), which points to the need to plan for and follow stringent rules for reinstitution of measures.

The social contact network used for this study was constructed to represent a small community in which complexity of person-to-person transmission can be represented in detail. Once within a community, influenza transmission takes place in the subnetworks simulated by this model, such as workplace, school groups, households, and neighborhoods. A larger population, such as a city, could be best modeled as a set of communities that are in contact through interactions in the work environment or through random interactions in shops or other settings. Mathematically, our model and results apply to such a situation where subnetworks in each community are similar, the epidemic is equivalently initiated in each, and identical mitigation strategies are applied with equivalent thresholds and compliances. Results then apply equally to single and large composite communities; rescinding thresholds should most conservatively be applied at the scale of the composite community.

The need to formulate regionally based policies for school closings has been suggested (*19*). For comparison, we simulated epidemics in which neighboring communities were not practicing the same community mitigation strategies. In these unmitigated regional simulations, workplace contacts were replaced by random contacts from a fully mixed reservoir of adults from neighboring communities doing nothing to abate the epidemic. When compared with mitigated regional epidemic scenarios, in mild or severe epidemics with child sequestering implemented, peak illness rates doubled and tripled, respectively. However, child sequestering still decreased illness attack rates by >75% from base cases. Community sequestering was a more effective strategy in unmitigated regional scenarios for mild and severe epidemics, keeping peak symptomatic cases equivalent to the mitigated regional scenarios, but it lost effectiveness with decreasing compliance. Thus, without regional practice of strategies, effectiveness was less, but the direction of effects was the same.

A recent US Institute of Medicine report properly describes modeling as a simplification of reality (*9*). The stylized community we modeled exemplifies that description. However, modeling is a useful way to explore ramifications of policy. This study outlines potential approaches for reopening schools and workplaces in a waning epidemic, but also points to additional resources required to apply them. Clear case definitions, diagnostic criteria, and availability of diagnostic tests would clarify strategy initiation triggers. Accurate community surveillance would be necessary to count cases and determine when a rescinding threshold had been reached and when measures might need to be reinstituted. Initiation and rescinding of community mitigation strategies could seem frustratingly complex to an unprepared public, pointing to the need for clear messaging and information dissemination about rationale and potential effectiveness of all mitigation measures.

Epidemic modelers and public health experts propose that community mitigation measures might help communities limit pandemic effects. Nonpharmaceutical, behavior-based measures could be critically important in the interval between availability of vaccine and arrival of antiviral medications. Our study builds on previous studies that examined timing and effectiveness of initiation of nonpharmaceutical pandemic mitigation measures by examining thresholds for rescinding them. We found that measures might be strategically applied and rescinded without the effect of additional illness and with savings of societal costs in terms of restriction of usual activities.

## Supplementary Material

Appendix Table 1 Outcomes of mild epidemics (PSI 1-2)*

Appendix Table 2 Outcomes of severe epidemics (PSI 4-5)*
