# Supplementary material for: Rescinding Community Mitigation Strategies in an Influenza Pandemic
Source: Emerg Infect Dis. 2008 Mar;14(3):365–72. doi: 10.3201/eid1403.070673 (PMC2570828; doi:10.3201/eid1403.070673)
Supplement: Appendix Table 1 — Outcomes of mild epidemics (PSI 1-2)* [file 07-0673_appT1.pdf]

Appendix Table 1. Outcomes of mild epidemics (PSI 1–2)*

Outcome	No. epidemics/100 simulations					Infection rate					Peak illness rate				
	97.2					0.496					0.084				
Unmitigated base case	Rescinding threshold					Rescinding threshold					Rescinding threshold				
	Cont	0	1	2	3	Cont	0	1	2	3	Cont	0	1	2	3
Child sequestering															
% Compliance															
90	**75**	**77**	**81**	**88**	**93**	**0.020**	**0.021**	**0.024**	**0.039**	**0.069**	**0.004**	**0.004**	**0.004**	**0.004**	**0.006**
80	**76**	**86**	**91**	**91**	97	**0.028**	**0.028**	**0.034**	**0.064**	**0.089**	**0.004**	**0.004**	**0.004**	**0.005**	**0.007**
70	**86**	**90**	**90**	**91**	98	**0.039**	**0.040**	**0.054**	**0.083**	0.113	**0.005**	**0.005**	**0.005**	**0.006**	**0.008**
60	**87**	**88**	**89**	**96**	99	**0.053**	**0.058**	**0.079**	0.100	0.144	**0.005**	**0.005**	**0.006**	**0.007**	**0.008**
50	**93**	**92**	**96**	**94**	97	0.100	**0.096**	0.103	0.135	0.171	**0.008**	**0.007**	**0.008**	**0.008**	0.010
Community sequestering															
% Compliance															
90	**65**	**50**	**61**	**75**	**89**	**0.016**	**0.017**	**0.020**	**0.027**	**0.036**	**0.00**	**0.00**	**0.00**	**0.00**	**0.01**
80	**78**	**76**	**82**	**90**	**92**	**0.019**	**0.021**	**0.029**	**0.041**	**0.065**	**0.00**	**0.00**	**0.00**	**0.01**	**0.01**
70	**82**	**83**	**84**	**93**	**94**	**0.025**	**0.026**	**0.035**	**0.057**	0.100	**0.00**	**0.00**	**0.00**	**0.01**	**0.01**
60	**89**	**94**	**92**	**92**	100	**0.037**	**0.038**	**0.051**	**0.078**	0.116	**0.01**	**0.00**	**0.01**	**0.01**	**0.01**
50	**96**	**90**	**96**	**94**	**95**	**0.061**	**0.067**	**0.078**	0.115	0.147	**0.01**	**0.01**	**0.01**	**0.01**	**0.01**

Outcome	Average no. cycles					Adult days at home					Average duration of strategy, d				
	0					2					0				
Unmitigated base case	Rescinding threshold					Rescinding threshold					Rescinding threshold				
	Cont	0	1	2	3	Cont	0	1	2	3	Cont	0	1	2	3
Child sequestering															
% Compliance															
90	**1**	**1.06**	**1.64**	2.86	5.75	12	**9**	**9**	**11**	15	54	**40†**	**38†**	**47**	64
80	**1**	**1.05**	**1.48**	3.48	5.43	15	**11**	**11**	17	18	66	**49†**	**50**	73	78
70	**1**	**1.08**	**1.8**	3.67	5.58	17	**14**	17	22	22	78	**64†**	**74**	97	96
60	**1**	**1.08**	**1.96**	3.49	6.37	20	**18**	22	24	28	92	**83†**	98	106	119
50	**1**	**1.07**	**1.76**	3.38	6.20	26	**25**	**24**	28	31	123	**113†**	**108**	125	131
Community sequestering															
% Compliance															
90	**1**	**1.1**	**1.52**	2.19	3.22	43	**29**	**28**	**29**	**29**	47	**32†**	32	32	33
80	**1**	**1.05**	**1.68**	2.72	4.95	41	**28**	**35**	**39**	47	51	**36†**	44	49	59
70	**1**	**1.06**	**1.68**	3.16	6.56	44	**33**	**37**	**48**	63	62	**47†**	52	68	89
60	**1**	**1.04**	**1.62**	3.43	6.45	47	**36**	**41**	53	61	78	**61†**	68	88	101
50	**1**	**1.04**	**1.81**	3.49	6.36	50	**45**	**48**	55	62	100	**90†**	96	110	124

*PSI, pandemic severity index; rescinding threshold, strategy ends when 0, 1, 2, or 3 new cases occur in 7 days (2× the generation time of influenza); Cont, strategy continuation for the duration of the epidemic. Values in **boldface** meet targets in the Table. Averages are for 100 simulations.

†Meets all 6 targets in the Table superimposed on shortest duration of strategies.
