# Supplementary material for: Rescinding Community Mitigation Strategies in an Influenza Pandemic
Source: Emerg Infect Dis. 2008 Mar;14(3):365–72. doi: 10.3201/eid1403.070673 (PMC2570828; doi:10.3201/eid1403.070673)
Supplement: Appendix Table 2 — Outcomes of severe epidemics (PSI 4-5)* [file 07-0673_appT2.pdf]

Appendix Table 2. Outcomes of severe epidemics (PSI 4–5)*

Outcome	No. epidemics per 100 simulations					Infection rate					Peak illness rate				
Unmitigated base case	100					0.714					0.170				
	Rescinding threshold					Rescinding threshold					Rescinding threshold				
	Cont	0	1	2	3	Cont	0	1	2	3	Cont	0	1	2	3
Child sequestering															
% Compliance															
90	100	100	100	100	100	0.176	0.173	0.196	0.243	0.277	0.014	0.013	0.013	0.014	0.016
80	**99**	100	100	**99**	100	0.364	0.372	0.375	0.389	0.400	0.033	0.034	0.034	0.035	0.034
70	100	100	100	**99**	100	0.461	0.461	0.465	0.461	0.469	0.052	0.052	0.052	0.052	0.053
60	100	100	**99**	100	100	0.512	0.514	0.514	0.511	0.517	0.065	0.066	0.065	0.064	0.066
50	100	100	100	100	100	0.563	0.556	0.558	0.561	0.561	0.080	0.079	0.079	0.080	0.079
Community sequestering															
% Compliance															
90	100	**98**	**99**	**97**	100	**0.045**	**0.046**	**0.063**	**0.095**	0.136	**0.007**	**0.007**	**0.007**	**0.008**	0.010
80	100	100	**99**	100	100	0.161	0.161	0.178	0.221	0.261	0.013	0.013	0.013	0.015	0.016
70	100	100	100	100	100	0.326	0.319	0.336	0.341	0.363	0.030	0.030	0.031	0.030	0.030
60	**99**	100	**99**	100	100	0.425	0.423	0.430	0.433	0.443	0.048	0.047	0.048	0.048	0.049
50	**99**	100	100	100	100	0.503	0.505	0.506	0.506	0.512	0.067	0.066	0.066	0.067	0.067

Outcome	Average no. cycles					Adult days at home					Average duration of strategy, d				
Unmitigated base case	0					3					0				
	Rescinding threshold					Rescinding threshold					Rescinding threshold				
	Cont	0	1	2	3	Cont	0	1	2	3	Cont	0	1	2	3
Child sequestering															
% Compliance															
90	**1**	**1.05**	**1.8**	3.82	5.43	28	**27**	31	38	38	132	**124**	138	165	163
80	**1**	**1.05**	**1.52**	2.39	4.15	26	**23**	**25**	26	28	133	**111**	**111**	**116**	**121**
70	**1**	**1.03**	**1.29**	**1.79**	2.99	22	**20**	**20**	**20**	**21**	116	**95**	**91**	**89**	**87**
60	**1**	**1.01**	**1.2**	**1.43**	2.48	20	**18**	**18**	**18**	**19**	107	**86**	**80**	**79**	**74**
50	**1**	**1.00**	**1.11**	**1.26**	**1.8**	19	**17**	**17**	**17**	**17**	101	**80**	**75**	**70**	**67**
Community sequestering															
% Compliance															
90	1	**1.02**	**1.78**	2.98	4.95	63	**49**	**58**	74	91	70	**54†**	**65†**	83	101
80	1	**1.05**	**1.65**	3.52	5.64	102	**88**	**96**	114	125	127	**111**	**119**	143	156
70	1	**1.05**	**1.54**	2.53	4.79	87	**77**	**77**	**82**	88	124	**109**	**110**	**117**	126
60	1	**1.02**	**1.29**	2.1	3.46	68	**56**	**55**	**55**	**53**	113	**93**	**91**	**91**	**89**
50	1	**1.01**	**1.23**	**1.42**	2.19	51	**43**	**40**	**37**	**36**	102	**86**	**79**	**74**	**71**

*PSI, pandemic severity index; rescinding threshold, strategy ends when 0, 1, 2, or 3 new cases occur in 7 days (2× the generation time of influenza); Cont, strategy continuation for the duration of the epidemic. Values in **boldface** meet targets in the Table. Averages are for 100 simulations.

†Meets all 6 targets in the Table superimposed on shortest duration of strategies.
